# Relationship between conservation biology and ecology shown through machine reading of 32,000 articles

**DOI:** 10.1111/cobi.13435

**Published:** 2019-12-10

**Authors:** Rogier E. Hintzen, Marina Papadopoulou, Ross Mounce, Cristina Banks‐Leite, Robert D. Holt, Morena Mills, Andrew T. Knight, Armand M. Leroi, James Rosindell

**Affiliations:** ^1^ Department of Life Sciences Imperial College London Silwood Park campus, Buckhurst Road Ascot Berkshire SL5 7PY U.K.; ^2^ Groningen Institute for Evolutionary Life Sciences University of Groningen Groningen 9747 AG The Netherlands; ^3^ Department of Biology University of Florida Gainesville FL 32611 U.S.A.; ^4^ Arcadia Fund Sixth Floor, 5 Young Street London W8 6EH U.K.

**Keywords:** bibliometrics, ecological applications, ecological theory, interdisciplinary, latent Dirichlet allocation, aplicaciones ecológicas, asignación latente Dirichlet, bibliometría, interdisciplinario, teoría ecológica, 潜在狄利克雷分布模型, 跨学科, 生态学理论, 生态学应用, 文献计量学

## Abstract

Conservation biology was founded on the idea that efforts to save nature depend on a scientific understanding of how it works. It sought to apply ecological principles to conservation problems. We investigated whether the relationship between these fields has changed over time through machine reading the full texts of 32,000 research articles published in 16 ecology and conservation biology journals. We examined changes in research topics in both fields and how the fields have evolved from 2000 to 2014. As conservation biology matured, its focus shifted from ecology to social and political aspects of conservation. The 2 fields diverged and now occupy distinct niches in modern science. We hypothesize this pattern resulted from increasing recognition that social, economic, and political factors are critical for successful conservation and possibly from rising skepticism about the relevance of contemporary ecological theory to practical conservation.

## Abstract 2

*Article impact statement*: Quantitative literature evaluation reveals that the research topics of ecology and conservation biology are drawing apart.

## Abstract (es)

Relaciones entre la Biología de la Conservación y la Ecología Mostradas a través de la Lectura Mediante Máquina de 32,000 Artículos

**Resumen**

La biología de la conservación se fundó a partir de la idea de que los esfuerzos para salvar a la naturaleza dependen del entendimiento científico de cómo funciona. La biología de la conservación buscaba aplicar los principios ecológicos a los problemas de conservación. En este trabajo investigamos si la relación entre estos ámbitos ha cambiado con el tiempo al realizar una lectura mediante máquina de 32,000 textos completos de artículos de investigación publicados en 16 revistas sobre ecología y biología de la conservación. También examinamos los cambios en los temas de investigación en ambos ámbitos y cómo éstos han evolucionado desde el año 2000 hasta el 2014. Conforme ha madurado la biología de la conservación, su enfoque se ha movido de los aspectos ecológicos de la conservación a los aspectos políticos y sociales. La ecología y la biología de la conservación se han separado y ahora ocupan nichos distintos dentro de la ciencia moderna. Nuestra hipótesis considera que este patrón resultó de incrementar el reconocimiento de que los factores sociales, económicos y políticos son muy importantes para una conservación exitosa. Posiblemente el patrón también proviene del creciente escepticismo acerca de la relevancia que la teoría ecológica contemporánea tiene para la conservación en práctica.

## 摘要

保护生物学是建立在拯救自然的努力是依靠对自然过程的科学理解的基本理念之上, 它致力于将生态学原理应用于保护问题。我们通过对发表在 16 个生态学和保护生物学期刊上的 32,000 篇研究论文全文进行机器阅读, 分析了这两个领域的关系是否随时间发生了变化。本研究涵盖了 2000‐2014 年间这两个领域研究主题的变化及其演变。结果显示, 随着保护生物学学科成熟, 它的关注点从生态学转向了保护的社会和政治方面。因此, 保护生物学与生态学也逐渐分歧, 并在现代科学中占据了截然不同的位置。我们认为这种格局是由于越来越多的人意识到社会、经济和政治因素对保护成功与否至关重要, 也可能是由于越来越多的人开始怀疑当代生态学理论与保护实践之间的联系。**【翻译: 胡怡思; 审校: 聂永刚】**

## Introduction

Conservation biology was born of a union between ecology and the ethical impulse to preserve its object, the world of living things. Pioneering ecologists (von Humboldt & Bonpland 1819; Wallace 1869) and many others since have seen its destruction clearly. Some became public defenders of biodiversity; others ran organizations devoted to doing so; many applied the lessons of their science to conservation. Though the relationship between science and advocacy has often been fraught (Worster 1994; Cafaro & Primack 2014; Kareiva et al. 2014), it is clear to most ecologists that natural diversity demands not merely curiosity, but protection. This is so in good part due to Michael Soulé’s founding of a new academic field, conservation biology (Van Dyke 2008). Soon after, a new scholarly society and a new journal—this one—were founded. Both have flourished. Like cancer biology, he said, conservation biology should rest on scientific and normative principles. It was about applying ecology and evolutionary biology to the goal of conserving nature (Soulé 1985). Underscoring the urgency of its task, he spoke of conservation biology as a “mission‐oriented,” “crisis” science. The Society for Conservation Biology now has thousands of members and chapters across the globe (Meine et al. 2006). At least 24 journals are now largely devoted to conservation biology (Bradshaw & Brook 2016). Conservation biology education programs proliferate.

Conservation biology is now a mature field. By some metrics it is larger than ecology. Its parentage is visible in the many conservation articles based on concepts from ecology and evolutionary biology. Island biogeography and metapopulation dynamics fueled discussions about the design and size of nature reserves (May 1975; Hanski 1989); stochastic population dynamics models gave rise to population viability analysis (Shaffer 1981); and population genetics warned of the risks of inbreeding depression (Soulé & Simberloff 1986). But this list has an old‐fashioned feel. The theory behind these examples dates to the mid‐20^th^ century; they are the founding principles of the field (Soulé & Wilcox 1980) and remain the most important ecological principles in conservation despite the many theoretical advances and empirical results that have transformed ecology over the last 40 years (e.g., May 1972; Chesson 2000b; Bell 2001; Hubbell 2001). We hypothesize that ecology's role in conservation biology has waned and that the vision of a science that applies the latest ecological ideas to solving its pressing problems has faded too.

We could have tried to test this hypothesis with a traditional literature review. Instead, we did so through primary research with text‐mining tools. We applied machine reading to the full texts of articles published in top ecology and conservation biology journals from 2000 to 2014. Using these data, we constructed a map to identify research topics that are largely distinctive to ecology and conservation biology and those they have in common. We studied how attention to these topics has changed over time, and by constructing a citation network, we examined the topics in each field that have most influenced the other. We also considered how conservation biology and ecology might strengthen their relationship.

## Methods

### Topic Analyses

We used a class of unsupervised machine learning known as topic analysis. Topic analysis rests on the idea that any document in a corpus can be characterized by its mix of topics that comprise groups of statistically associated words. Specifically, we used latent Dirichlet allocation (LDA) (Blei et al. 2003) as our topic model. This algorithm estimates for each word the probability of belonging to each topic and for each document the probability that a random word originated from a given topic. Together, these reveal what an article is about.

As an example, we ran a 2‐topic LDA model on our corpus of 32,104 conservation and ecology articles. The words found with the highest probabilities in each topic are highlighted in Fig. b. Where the most probable words in topic 1 related to conservation biology, and those in topic 2 related to ecology. We did not ask the algorithm to find these particular topics; rather, it discovered them by itself. We did, however, tell it how many topics to discover.

**Figure 1:**
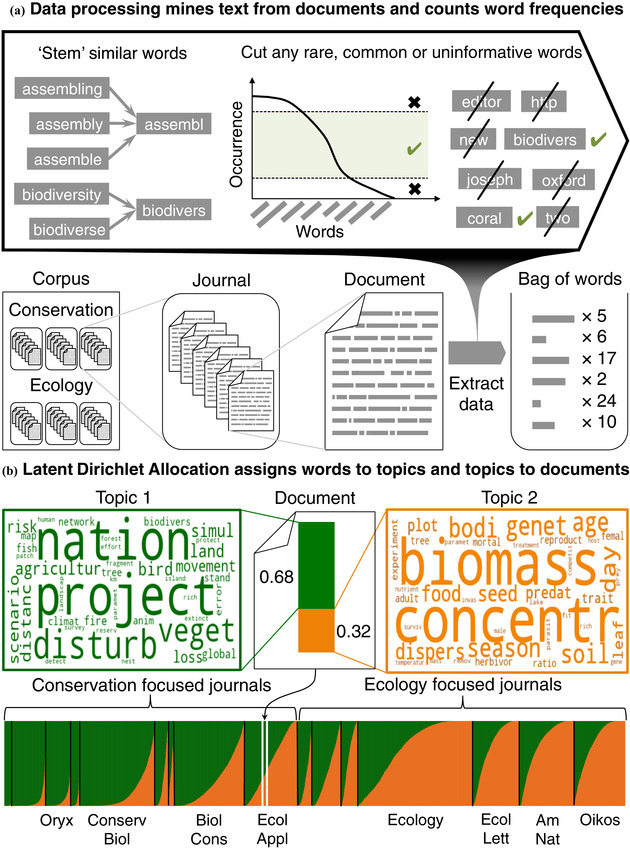
Topic modeling overview and example: (a) data‐processing overview showing the structure of the data set (corpus of articles published in ecology and conservation biology) and the methods applied to it (each article reduced to word frequencies after filtering for very rare and very common words) and (b) the example topic model assigns a probability that each of k topics is found in a given article and that each word is found in a given topic. Topics are interpreted by examining the top n most probable words as represented by word clouds in which the size of a word is scaled relative to its probability. This example shows the results of a 2‐topic model in which topic 1 represents a general conservation topic and topic 2 a general ecology topic. The nature of these topics is reflected in their distribution among the individual articles in the 16 journals in the corpus.

An LDA is technically identical to the finite admixture model in STRUCTURE (Pritchard et al. 2000). The only real difference between them is that one analyzes the structure of words present in a population of documents, whereas the other analyzes the structure of genetic variants in a population of organisms (similarity illustrated in Fig. b). Although population geneticists generally assume genetic structure depends on geography, we began with the idea that lexical structure depends on field (i.e., an abstract geography in the space of the mind).

### Constructing and Understanding the Corpus

We asked experts (workshop attendees) which journals were most important in ecology and conservation biology. Sixteen journals were identified (general journals, such as *Nature* and *Science*, were excluded; Supporting Information), nearly all of which are among the most highly ranked in their fields (Bradshaw & Brook 2016). We classified our journals as ecology or conservation biology based on the opinions of attendees of the workshop and some others, the aims‐and‐scope statements of the journals, and SCOPUS keyword tags. These criteria consistently identified our 16 journals as belonging primarily to one or the other field.

We used GNU Wget version 1.17.1 to download the full text of all available articles directly from the journal websites. The resulting database consisted of 32,104 articles distributed across the 16 journals. The corpus contained 16,639 articles on ecology and 15,465 articles on conservation biology. Using Python text‐mining tools, we obtained the full text of each article, including its abstract, publication year, and journal name, but not bibliographies. Using the SCOPUS database as an independent count of the number of articles in each journal, we estimated that we obtained >95% of the articles published in the 16 journals from 2000 to 2014.

To determine article topics, we constructed a vocabulary. Using the Natural Language Toolkit in Python (Loper & Bird 2002), we obtained a list of all words they contained. We stemmed the words (reducing all inflected words to a common root) and removed both rare (in <0.1% of articles) and common (in >80% of articles) words as uninformative. The remaining 7686 unique words constituted the corpus’ vocabulary.

Using this vocabulary, we modeled the structure of the corpus with LDA (Blei et al. 2003), as implemented in the Python package genism (Řehůřek & Sojka 2011). The LDA model requires choosing the number of topics, *k*. To determine the optimal number of topics, we ran models with 5 ≤ *k* ≤ 300 and assessed their goodness of fit with topic coherence (Röder et al. 2015). We used the optimal model *k* = 50 (Supporting Information) in all subsequent analyses. We labeled the topics by assessing the top 50 most common words and the most salient words for each topic in the PyLDAvis package (Supporting Information). Analysis steps are summarized in Fig. 1. To confirm that our model described the corpus, we tested it with 2 of the most cited articles in our corpus: Gotelli and Colwell (2001) published in *Ecology Letters* (4978 citations in Google Scholar) and Debinski and Holt (2001) published in *Conservation Biology* (1,486 citations).

### Exploring Topic Space

To understand the contents of our 16 journals, we compared the average (median log_10_[topic probability]) distributions for each, clustering both topics and journals with Hellinger distance. We constructed a landscape of ecology and conservation biology by using *t*‐distributed stochastic neighbor embedding (*t*‐SNE) to reduce the 50‐topic dimensions to 2 (Maaten & Hinton 2008; Pedregosa et al. 2011) and estimated the probability density of articles in this 2‐dimensional space with a Gaussian smoothing kernel. This map is a 3‐dimensional landscape in which the peaks and valleys represented the relative number of articles found at any point. Each point was a unique combination of topics, but peaks tended to be formed from articles rich in particular topics. To visualize the evolution of the fields, we divided articles a priori into ecology and conservation biology fields and 3 periods by publication date (2000–2004, 2005–2009, and 2010–2014). We also estimated the Shannon diversity of topics within the 2 fields as a function of time.

### Identifying Field‐Biased Topics

We identified the topics most prevalent in each field relative to the other (i.e., field‐biased topics) from the full distribution of topic probabilities of all the documents in each field and from discretized topic probabilities based on a threshold. Both methods gave similar results.

To determine the full distribution of topic probabilities for each field, we calculated the median log_10_(probability) for each topic across all articles in that field. We then calculated the differences in all median probabilities among fields. Ecology‐biased topics are those in which this difference is positive; conservation‐biased topics are those in which it is negative (Supporting Information).

The precise probability of a topic in an article is not always informative. This is because all topics have a nonzero probability of appearing in all articles, but in some it is very small. To determine whether an article was about a topic, we discretized the probabilities by setting a threshold of 0.05. Only if a topic exceeded this probability was an article considered to be about that topic. Because articles can be about many topics, we estimated the probability that a random article from each field was about each topic. We calculated the ratio of these probabilities to get a relative risk (RR) of occurrence for each topic. We defined ecology‐biased topics as those with a high RR and conservation biology‐biased topics as low RR (Supporting Information).

Trends in median topic probability were modeled as a single quintile regression. Trends in discretized topics were modeled as linear models with second‐ and third‐order polynomial terms, and the most appropriate model was chosen with analysis of variance and Akaike information criterion.

### Obtaining and Mapping the Citation Network

Many of the full‐text articles were poorly structured, meaning we could not consistently obtain their titles, DOIs (direct object identifier), or bibliographies. We therefore obtained article titles, DOIs, and bibliographies from SCOPUS and used these data to build a citation network with the Diderot package (Vincenot 2017). Using the full‐text trained LDA model, we estimated the topic distributions of their abstracts and reestimated the probability density distributions for each field in *t*‐SNE space. When doing so, however, we weighted the densities by their relative propensity to be cited by articles belonging to the other field. Thus, we constructed separate landscapes for ecology and conservation articles whose peaks represented areas in each cited by the other field.

## Results

The highly cited test article Gotelli and Collwell (2001) was mostly about the topics of macroecology (38.2%), ecological research (13.1%), statistical inference (11%), monitoring biodiversity (10.5%), and assessing and managing extinction risk (8.2%). Debinski and Holt (2001) was mostly about the topics of habitat fragmentation (29.6%), assessing and managing extinction risk (10.2%), ecosystem function and response to change (8.7%), experimental ecology (7.3%), and ecological research (6.9%). We read the articles, and these descriptions were consistent with our assessments of the topics.

Our model indicated what we already knew about the contents of particular journals. The most frequent topic in *The American Naturalist*, home to many articles on estimating natural selection and inferring adaptations, was trait evolution (9%). The most frequent topics in *Conservation Biology* and *Biological Conservation* were conservation and society (13%) and managing and assessing extinction risk (10%), respectively.

**Figure 2:**
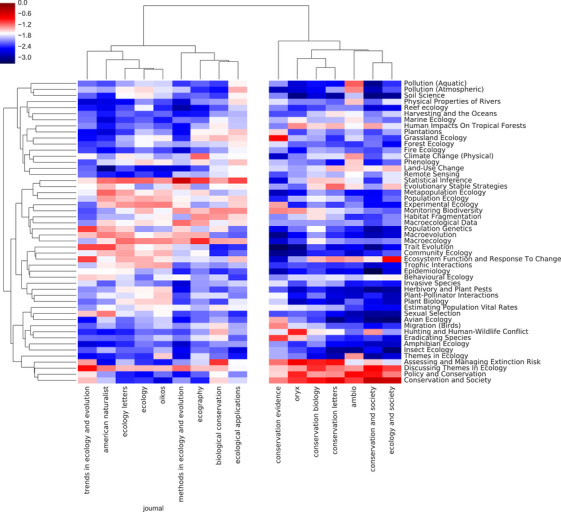
Topics in ecology and conservation journals and research programs as revealed through topic modeling. The heatmap shows the median log_10_(probability) assigned to each topic for each journal.

Journals we tagged a priori as conservation biology had high average probabilities for topics such as conservation and society, assessing and managing conservation risk, policy and conservation, and ecosystem function and response to change, whereas in the journals we tagged as ecology topics such as statistical inference, macroecology, experimental ecology, and trait evolution (Fig. 2) prevailed. Hierarchical clustering divided the journals into 2 groups that, with 2 exceptions, corresponded to our a priori classification. The 2 exceptions were *Ecological Applications* and *Biological Conservation*, which we assumed belonged in conservation biology but clustered with ecology. This inconsistency may reflect their true interdisciplinary nature.

**Figure 3:**
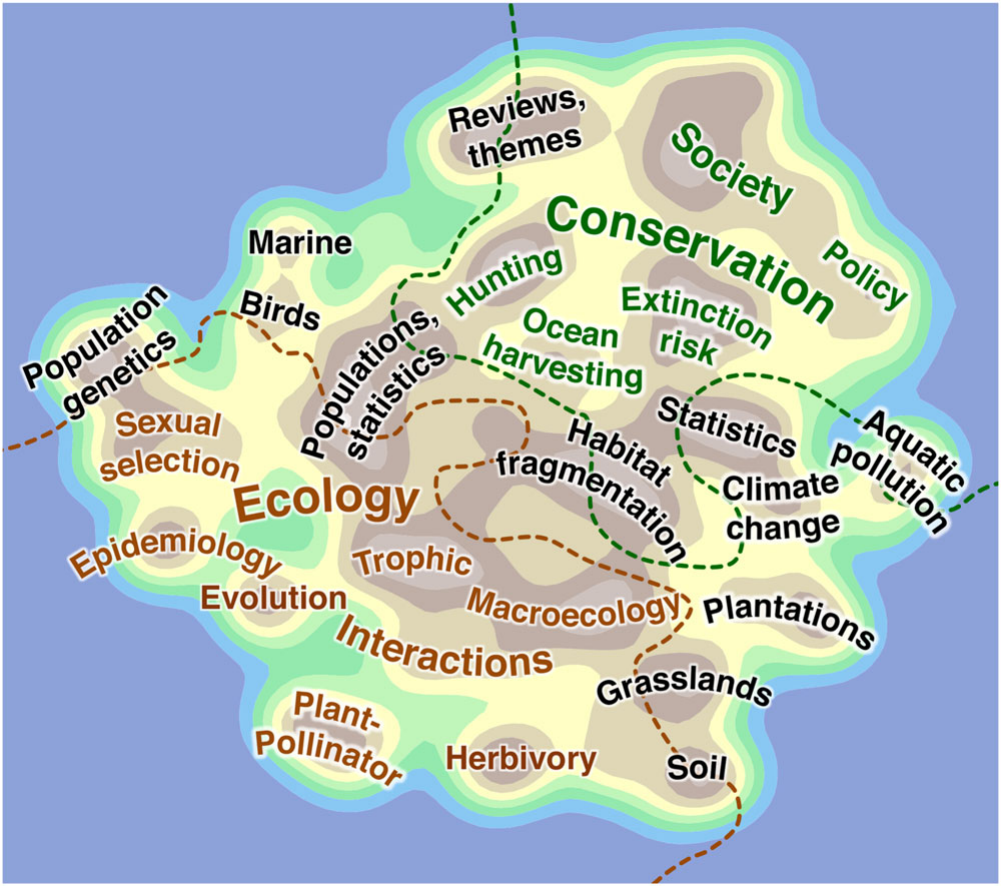
Distribution of the entire ecology and conservation biology corpus in t‐SNE space (*t‐*SNE, t‐distributed stochastic neighbor embedding) represented as a topographical map (peaks, where many articles lie, labeled based on dominant topics in the articles; valleys, where fewer areas articles lie; dotted line, boundary between fields based on mismatch in t‐SNE space [Supporting Information]).

The topographic map of the distribution of articles in 2‐dimensional topic space (Fig. 3) showed, for example, in the northeast a topic range for society and policy. In the southwest, there was a central ecological range that consisted of peaks corresponding to different kinds of species’ interactions. Articles about habitat fragmentation and climate change appeared between these topic areas.

This map must be read with care because *t*‐SNE, like any dimensionality reduction method, necessarily resulted in loss of information. The *t*‐SNE algorithm accurately models local distances in 2‐dimensional space at the cost of accuracy over longer distances. Similar articles are placed close together, but distances between very dissimilar articles may not be meaningful. Even so, we could discern the frontier between ecology and conservation based on the location of points where the articles were evenly distributed between the 2 fields (Supporting Information). The boundaries ran, with some diversions, from the northwest to southeast of our map, separating, for example, mount “habitat fragmentation” from the central ecological range. The boundary often followed valleys, indicating that the high‐elevation regions—those heavily populated by articles—typically originated in a single field. But the boundaries also sometimes traversed topographical features, where ecologists and conservation biologists have evidently found common ground, for example, in regions such as in the “population and statistics” massif and “bird” hill.

**Figure 4:**
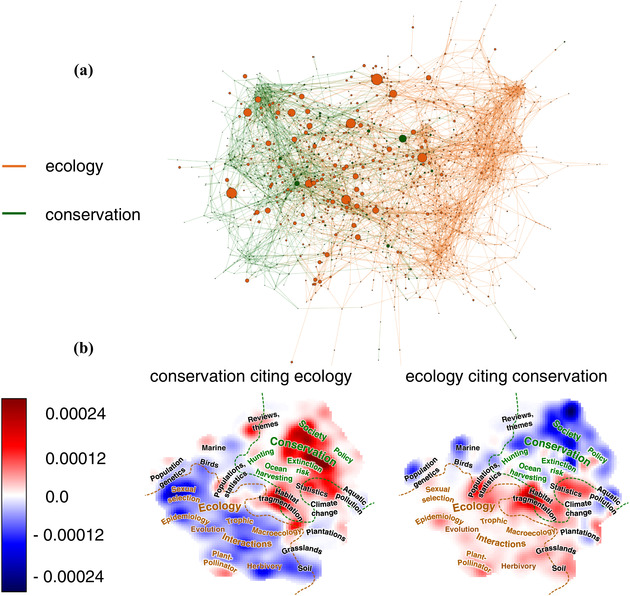
(a) Ecology and conservation biology citation network for the top 5% most cited articles (nodes [i.e., articles] scaled by number of cross‐citations received; lines, citation links) (b) Relative propensity of each field to be cited by the other (red, areas of topic space in each field that are highly cited by the other). For example, conservation biology articles tend to cite ecology articles relatively often when they are about extinction risk.

Of the 1,693,552 citations in our corpus, 136,946 were to articles also included in our data set (just over 8%). Of these, 76.7% of citations in ecology articles were to other ecology articles, and 86.7% of citations in conservation articles were to other conservation articles. But 26,476 citations crossed field boundaries: 23.3% of citations in ecology articles were to conservation articles, and 13.3% of citations in conservation articles were to ecology articles (Fig. 4). When peaks in the topic landscape were made proportional to number of citations attracted across the conservation—ecology boundary, the map showed that ecology articles were most likely to be cited in conservation biology articles when they were about core conservation topics (e.g., extinction risk) or when they were about topics that bordered the fields (e.g., habitat fragmentation) (Fig. 4). The reverse was also true. But there was also evidence of deeper cross‐field citation. For example, conservation articles also cited ecology articles about plant pollinator interactions, even though it is a core ecological topic. This may reflect a recent interest of conservation scientists in ecosystem services provided by pollinators, a topic that draws on knowledge of pollinator ecology. The layout algorithm placed nodes with a high density of links together. In general, this showed that although the citation networks of the 2 fields form 2 clearly distinct networks (Fig. 4), they were not completely isolated; a few areas of topic space encourage cross‐field citations and so a flow of ideas.

**Figure 5:**
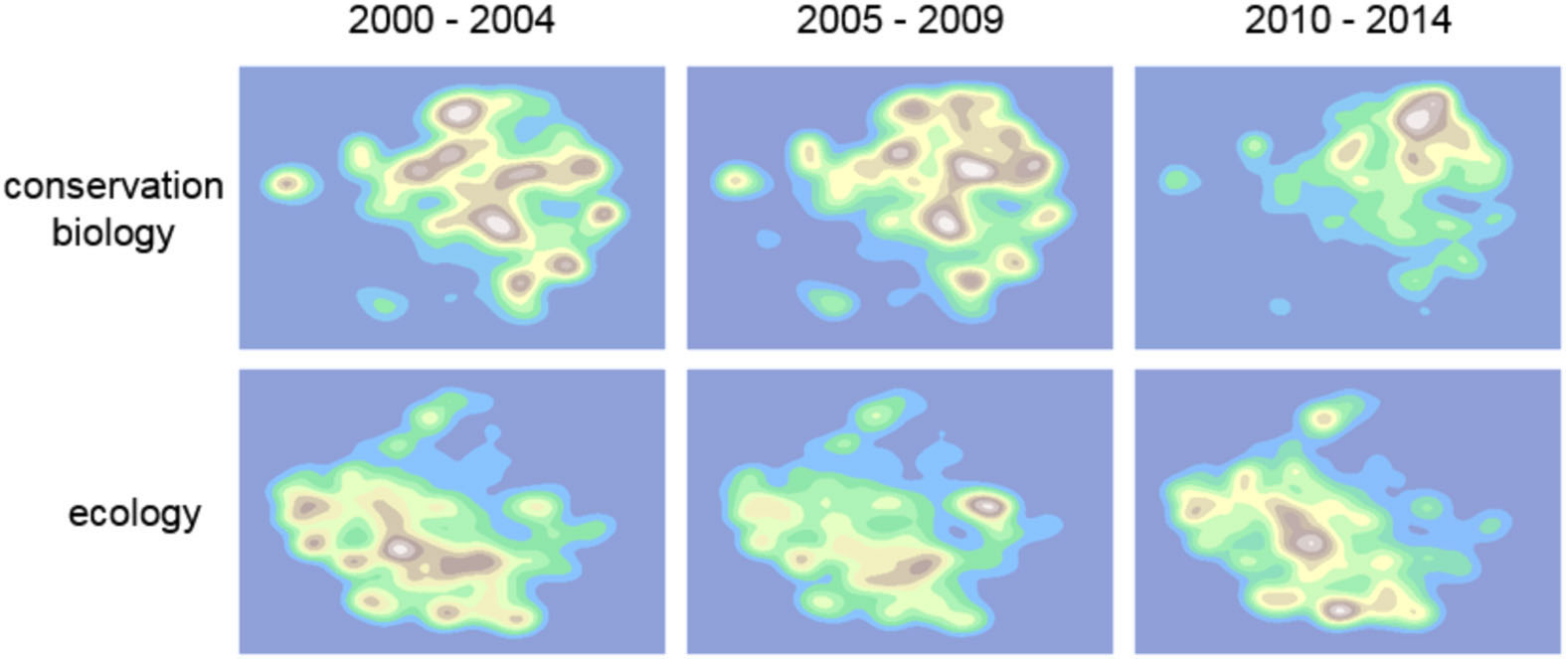
Change in focal interest in ecology and conservation biology from 2000 to 2014. Distribution of articles is in t‐SNE space (SNE, stochastic neighbor embedding).

But, disciplines evolve. We found that, while ecology's landscape has not changed much, conservation biology's has changed dramatically (Fig. 5). Peak society in the northeast quadrant of Fig. 5 is new. It began to build after 2005 and only arrived at its present form in the last decade. Its relative height suggests conservation biology, as measured by the distribution of topics, has become more homogeneous and increasingly concerned with the study of human behavior and the social factors and policies that influence it.

Thus, it seems conservation biology has become more specialized. To confirm this trend, we examined the evolution of field‐biased topics, which are more prevalent in one field than the other (e.g., trait evolution in ecology and national parks in conservation). We identified the 10 most biased topics for each field (Supporting Information) and examined the evolution of their summed probabilities in each field from 2000 to 2014. We modeled the data as a single linear quintile regression and found a highly statistically significant 3‐way interaction term (field:bias:year [Supporting Information]). The contribution of conservation‐biased topics to conservation articles increased (Fig. a) as the contribution of ecology‐biased topics declined (Fig. b). A plot of Shannon diversity of all topics in conservation biology articles showed a decline in diversity since 2005 (Fig. e). Ecology articles also became more homogeneous, but not as rapidly, indicating increased specialization.

**Figure 6:**
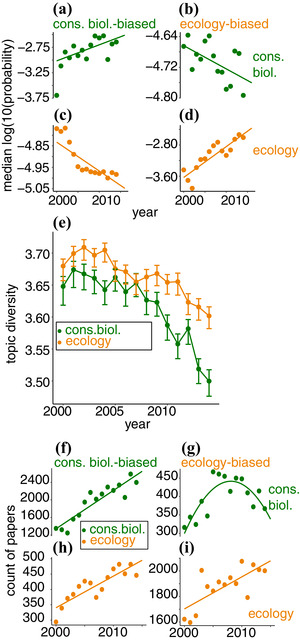
(a–d) Probability of topics favored (bias) in conservation or ecology being covered in articles from the other field. (e) Change in topic diversity in ecology and conservation biology over time (Shannon diversity based on discretized topic distributions; 95% CIs estimated by bootstrapping). (f–i) Change in absolute contribution of ecological and conservation ideas to ecology and conservation.

Both fields not only changed between 2000 and 2014, but also grew. If our corpus is a good indication, then the growth rates themselves also increased in that period (Supporting Information). This growth means (despite the decline in contribution of ecology‐biased topics to conservation) the absolute number of conservation biology articles with at least some ecological content may have remained constant or increased. We modeled the prevalence of ecological and conservation ideas in ecology and conservation journals with discretized topics and found that first‐order linear models gave the best fit in all cases except for the evolution of ecology‐biased topics in conservation. These were best described by a quadratic model with an intermediate peak that showed the initial rise and post‐2005 decline of the absolute importance of ecological ideas in conservation biology. This demonstrates that although the number of conservation articles with at least some ecology‐biased content increased from 2000 to 2005, this number later decreased so that by 2014 there was about as much ecology in conservation articles as a decade before (Fig. g) despite the growth in the field overall. Had the prevalence of ecology in conservation articles remained at its pre‐2005 levels, then our conservation journals would have contained about 1200 more articles with ecology‐biased topics than they actually did. By contrast, the number of ecology articles with at least some conservation‐biased content rose steadily (Fig. h), though at a lower rate than the number of articles with ecology‐biased content. Eight of the 10 ecology‐biased topics declined post‐2005 in conservation biology; only community ecology and trophic ecology kept pace with the growth of the field as a whole (Supporting Information). Ecology, then, has not just become relatively less important to conservation biology, but absolutely so.

## Discussion

With our text‐mining techniques the vastness of the conservation and ecology literature, previously so daunting, became an asset because it allowed us to formally test hypotheses about the evolution of scientific ideas. Of course, the cost of this wide view is loss of detail. Topic analysis is a blunt tool: it reveals roughly what an article is about but not whether its subject was commended or criticized. To find that out, one would have to read. Even so, we suggest that, from now on, review articles will increasingly rely on quantitative data rather than impression, however astute, and opinion, however wise. The study of science has now, itself, become a science (Fortunato et al. 2018).

Conservation biology was founded on the idea that the principles of organismal science can and should be applied to efforts to protect biodiversity (Soulé & Wilcox 1980; Soulé 1985). We found it has become much what Michael Soulé envisioned it might be. Unsurprisingly, community ecology and food webs have a place in its literature, but even unconventional subjects such as sexual selection do as well (e.g., Wedekind 2002). Our finding that conservation biology has its own concerns andcitation network shows not that it is isolated from ecology, but that it is mature.

Our results also show that from 2000 to 2014 the contribution of the social sciences to conservation biology became ever greater, even as ecology's contribution declined. The fields are drifting apart, with conservation moving farther from ecology than the reverse (Fig. 5). These trends are not just caused by the expansion of conservation biology into new areas of research because, over the last 10 years, there has also been an absolute decline of the prevalence of ecological ideas in conservation (Fig. 6). What has driven these changes? We estimate that over 1000 ecology‐rich articles have gone in a way missing from conservation journals since 2005. One possibility is that they were written and published, but not in the journals we examined. Because new journals are being founded all the time, we cannot eliminate this explanation, but we think it is implausible. Our 9 conservation biology journals are the most important in the field. Four of them were founded after 2000, none of them has folded, and only one—discussed below—has had an obvious (very recent) change in remit. It seems most likely, then, that the missing articles were not written. At the least, we can say that if the missing articles were published, they were published in less highly regarded journals, thus still indicating a decline in interest in ecology.

A more subtle possibility is that the observed trends are the result of selection due to journal specialization. We imagine authors deciding whether to send a manuscript to the fictitious journals *Open Ecology* or to *Current Conservation*. They decide it is more likely to be accepted by the latter and so rewrite it, soft‐pedalling its implications for ecology but emphasizing those for conservation. The manuscript is accepted, is widely read and imitated, and so a consensus emerges among authors, reviewers, and editors that *Current Conservation* is not the place to sell grand theory. If this process is at work, then it is journals, not scientists, that have become more specialized, but without analyzing articles by author, one cannot know whether this is so. Either way, the result is the same:2 diverging literatures. And the question remains: given that scientists and editors are rational agents who aim to produce and publish the most exciting possible research, if applying ecological theory to conservation is such a great idea, then why are articles that do so declining in the field's major journals?

One answer is that the founding promise of conservation biology—that ecological theory could help us save organic diversity—has not yet been fulfilled. The history of ecological ideas in conservation biology provides abundant evidence for this. Ecologists have often suggested how their ideas might be applied to conservation problems only to discover that they are not that useful after all. Take, for example, the power‐law species–area relationship (SAR), which is so ubiquitous that many speak of it as a law (Lawton 1999; Lomolino 2001). The SAR has accordingly been used to predict the risk of species extinction due to habitat loss (Desmet & Cowling 2004; Axelsen et al. 2013; Rybicki & Hanski 2013). But its ability to do so turns out to be very limited (Lewis 2006; He & Hubbell 2011; Stein et al. 2014). It is, then, a shaky foundation for a conservation strategy.

Even when ecological models are, in principle, applicable to conservation, they can be cumbersome to apply. Network and food‐web theory, which aim to elucidate how ecosystem stability depends on the diversity of and pattern of interactions among species (May 1973; Tilman et al. 2006; Jacquet et al. 2016), promised to predict the responses of ecosystems to perturbation (Urban et al. 2016). But it turns out ecological networks are hard to build and quantify, much less apply to any particular conservation problem (Thompson et al. 2001; Tylianakis et al. 2008). Once again, the promise of theory has not been fulfilled.

Such examples could be multiplied. Recent developments in ecology are no more promising. Modern coexistence theory, for example, describes the mechanisms that allow stable, diverse assemblages of coexisting species (Chesson 2000a; Barabás et al. 2018). It seems that coexistence theory might be useful in cases where the population declines of particular species are not caused directly by persecution, but rather by the indirect effects of other anthropogenic changes. Despite this—and though it is nearly 20 years old—coexistence theory barely features in conservation biology (Chapron & López‐Bao 2016). We suspect this is because it is technically complex (Pásztor et al. 2016; Barabás et al. 2018) and the equalizing and stabilizing factors upon which the theory depends can scarcely be measured in the wild.

Of course, it may be that modern ecological theories will prove useful in the future. Theory is often far in advance of empirical studies and they, in turn, often precede practical use. Pauli postulated the existence of neutrinos in 1930, yet they were only identified in 1956, and only recently has anyone found a use for them (e.g., Stancil et al. 2012). Perhaps the time lag between ecological theory and conservation is just as long. If so, it would be good to shrink it.

Beyond the difficulty of applying particular theories, there is a profound difference between the goals of ecology and conservation. Ecology has traditionally sought to understand the processes that govern populations, communities, and ecosystems at or near equilibrium (Brussard 1991, but see Hastings et al. 2018). But conservation biology is, by definition, concerned with those parts of nature that are in a state of flux. Moreover, conservation strategies necessarily entail predicting the future, and ecological forecasting is notoriously hard (Godfray & May 2014; Petchey et al. 2015).

The goals of conservation and ecology differ in a less abstract way too. While ecologists work at elucidating the great patterns of biodiversity and their causes, conservation scientists are busy trying to preserve biodiversity with the few resources they have (Bawa et al. 2004). And conservation biologists need to balance conserving species with human welfare. The importance of doing so is behind the rise (that we document) of research concerned with how humans affect nature and how those interactions might be altered for the better. Social science has been part of conservation biology since its birth (Soulé 1985), but Bennett et al. (2017) recently captured its increasing importance when they identified conservation social science as a new subfield. This journal has since offered its articles a home (Teel et al. 2018). Conservation is, after all, an argument among people.

These, we suggest, are some of the reasons conservation scientists have recently turned from ecological theory to research programs that promise more practical results. We do not believe that ecology in the broadest sense will go extinct in conservation biology. Ecological studies of threatened species will always be needed (Brussard 1991); new techniques, directly applicable to particular problems, will continue to be used. Population genomics, movement ecology, and infectious disease ecology in particular seem to have obvious conservation applications (Brannelly et al. 2018; Fraser et al. 2018; O'Hanlon et al. 2018). Yet it seems to us that the decline of general ecological ideas in conservation is both real and worrying.

Ecologists often claim that the patterns in nature they discover, and their explanations for them, are of vital importance to conservation. They do so in their grant proposals just as cell biologists assure reviewers that their research will surely save lives. Cell biologists have learned a lot about the molecular causes of disease. But improvements in cancer survival rates have been mostly due to more mundane things, such as incremental improvements in detection and surgical techniques and smoking's decline. In the same way, improvements in conservation practice are less due to a deeper grasp of ecological mechanisms than a better understanding of which piecemeal conservation strategies actually work (e.g., Sutherland 2018).

A cynic might suppose that because cancer still kills, the resources devoted to cell biology research were misspent. However, recent advances in immunotherapy suggest cynics will be proved wrong. So, we remain optimistic that new kinds of ecological theory will eventually prove their practical worth. We are thinking, in particular, of mechanistic models of biodiversity. Such models are based on ecological principles, require little data for parameterization, and may permit transparent, general, and useful ecological forecasts (Harfoot et al. 2014; Petchey et al. 2015; Urban et al. 2016). They are still new, but we think they are one reason among many that ecology and conservation biology may find that they need each other after all.

## Supporting information

A list of journals (Appendix S1), model coherence (Appendix S2), top 15 words per topic (Appendix S3), field‐specific bias of topics (Appendix S4 & S5), difference between conservation biology and ecology independently calculated kernel density estimation (Appendix S6), modeling change in topic probabilities (Appendix S7), number of articles by year (Appendix S8), number of conservation articles by topic (Appendix S9), and Fig. 6 with a unified y‐axis scale (Appendix S10) are available online. The authors are solely responsible for the content and functionality of these materials. Queries (other than absence of the material) should be directed to the corresponding author.
