# Supplementary material for: Relationship between conservation biology and ecology shown through machine reading of 32,000 articles
Source: Conserv Biol. 2019 Dec 10;34(3):721–32. doi: 10.1111/cobi.13435 (PMC7317371; doi:10.1111/cobi.13435)
Supplement: Supplementary file 1 — A list of journals (Appendix S1), model coherence (Appendix S2), top 15 words per topic (Appendix S3), field‐specific bias of topics (Appendix S4 & S5), difference between conservation biology and ecology independently calculated kernel density estimation (Appendix S6), modeling change in topic probabilities (Appendix S7), number of articles by year (Appendix S8), number of conservation articles by topic (Appendix S9), and Fig. 6 with a unified y‐axis scale (Appendix S10) are available online. The authors are solely responsible for the content and functionality of these materials. Queries (other than absence of the material) should be directed to the corresponding author. [file COBI-34-721-s001.docx]

**Supporting Information**

**S1**: The journals we studied, the number of papers we obtained from each, the years they were published in, and the field to which we assigned them *a priori*.

| journal | papers | years | field |
| --- | --- | --- | --- |
| *Ecography* | 1454 | 2000–2014 | ecology |
| *Methods in Ecology and Evolution* | 572 | 2010–2014 | ecology |
| *American Naturalist* | 2517 | 2000–2014 | ecology |
| *Ecology Letters* | 2105 | 2000–2014 | ecology |
| *Ecology* | 5067 | 2000–2014 | ecology |
| *Trends in Ecology and Evolution* | 1530 | 2000–2014 | ecology |
| *Oikos* | 3394 | 2000–2014 | ecology |
| *Ecological Applications* | 2586 | 2000–2014 | conservation biology |
| *Ambio* | 1455 | 2000–2014 | conservation biology |
| *Ecology and Society* | 1395 | 2000–2014 | conservation biology |
| *Conservation Biology* | 3531 | 2000–2014 | conservation biology |
| *Conservation Letters* | 410 | 2008–2014 | conservation biology |
| *Biological Conservation* | 4369 | 2000–2014 | conservation biology |
| *Oryx* | 1083 | 2000–2014 | conservation biology |
| *Conservation Evidence* | 270 | 2004–2014 | conservation biology |
| *Conservation and Society* | 366 | 2003–2014 | conservation biology |

**S2**: Coherence of Topic models


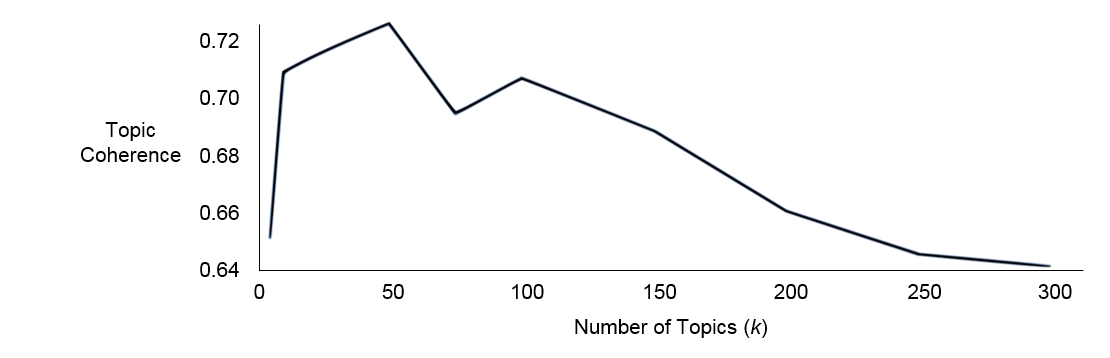


**S3:** All 50 topics with their top 15 most probable words and the assigned labels.

| topic | top 15 words |
| --- | --- |
| Human Impacts On Tropical Forests | forest, tropic, log, deforest, plantat, ha, tropic_forest, secondari, vol, dri, fragment, lowland, palm, rainforest, tree |
| Community Ecology | competit, interact, communiti, resourc, network, nich, coexist, competitor, structur, abund, mechan, posit, domin, pattern, facilit |
| Plant-Pollinator Interactions | seed, plant, flower, pollin, fruit, pollen, visit, seedl, bee, germin, dispers, nectar, product, reproduct, set |
| Trophic Interactions | predat, prey, food, consum, forag, trophic, web, feed, resourc, densiti, diet, food_web, abund, respons, biomass |
| Hunting and Human-Wildlife Conflict | park, hunt, wildlif, popul, deer, anim, eleph, bear, home, human, kill, nation, wild, home_rang, tiger |
| Metapopulation Ecology | dispers, model, extinct, dynam, rate, local, simul, paramet, equilibrium, colon, persist, assum, patch, valu, metapopul |
| Sexual Selection | femal, male, sex, reproduct, mate, offspr, sexual, ratio, size, matern, male_femal, sex_ratio, success, produc, condit |
| Invasive Species | invas, nativ, invad, exot, introduc, establish, plant, success, spread, introduct, alien, impact, propagul, control, non-nat |
| Amphibian Ecology | pond, wetland, amphibian, tadpol, frog, mesocosm, pool, aquat, water, salamand, toad, tank, terrestri, metamorphosi, habitat |
| Estimating Population Vital Rates | surviv, age, adult, mortal, year, life, juvenil, stage, reproduct, recruit, estim, rate, popul, histori, releas |
| Pollution (Aquatic) | lake, water, sediment, concentr, baltic, tion, organ, phytoplankton, sampl, depth, zooplankton, baltic_sea, pollut, surfac, benthic |
| Climate Change (Physical) | climat, region, climat_chang, temperatur, arctic, distribut, elev, global, model, warm, predict, environment, latitud, northern, precipit |
| Land-Use Change | land, agricultur, crop, farm, manag, product, servic, landscap, restor, field, ecosystem, cultiv, ha, region, loss |
| Eradicating Species | island, trap, vole, mammal, small, rodent, popul, anim, captur, lizard, snake, predat, fox, rat, activ |
| Statistical Inference | model, estim, variabl, valu, predict, distribut, paramet, set, method, sampl, observ, approach, error, simul, statist |
| Ecosystem Function and Response To Change | ecosystem, system, ecolog, process, function, scale, dynam, structur, exampl, respons, interact, impact, complex, understand, approach |
| Grassland Ecology | veget, plot, plant, graze, cover, grass, grassland, shrub, ment, site, year, soil, domin, communiti, type |
| Experimental Ecology | treatment, experi, experiment, control, test, day, respons, remov, interact, signific, measur, replic, anova, cm, manipul |
| Physical Properties of Rivers | water, river, stream, basin, flow, catchment, watersh, hydrolog, riparian, discharg, reach, flood, channel, dam, drainag |
| Epidemiology | host, parasit, infect, diseas, pathogen, transmiss, preval, immun, strain, suscept, virus, popul, spore, mite, virul |
| Fire Ecology | disturb, fire, burn, recoveri, regim, sever, year, frequenc, drought, fuel, recov, manag, respons, event, intens |
| Ecological research | ecolog, exampl, research, case, problem, question, make, review, work, discuss, interest, point, clear, approach, part |
| Pollution (Atmospheric) | nutrient, carbon, product, nitrogen, concentr, rate, ecosystem, ratio, measur, deposit, valu, biomass, emiss, estim, atmospher |
| Behavioural Ecology | group, anim, behaviour, behavior, risk, prefer, activ, respons, signific, social, inform, observ, signal, influenc, type |
| Population Ecology | popul, rate, densiti, growth, size, growth_rate, dynam, depend, demograph, variat, year, estim, paramet, model, declin |
| Habitat Fragmentation | habitat, patch, landscap, fragment, edg, size, connect, type, distanc, scale, qualiti, isol, spatial, variabl, suitabl |
| Harvesting and the Oceans | fish, fisheri, marin, mussel, stock, catch, crab, trout, salmon, mangrov, spawn, manag, biomass, shrimp, freshwat |
| Population Genetics | popul, genet, genotyp, gene, sequenc, allel, hybrid, sampl, dna, isol, divers, clone, loci, frequenc, genom |
| Avian Ecology | nest, egg, clutch, hatch, success, brood, size, predat, nestl, day, lay, chick, femal, reproduct, clutch_size |
| Macroevolution | size, bodi, phylogenet, mass, relationship, bodi_size, rate, length, signific, bodi_mass, test, analys, pattern, taxa, phylogeni |
| Reef ecology | reef, coral, obser, obser_ved, marsh, coral_reef, recruit, alga, cover, seagrass, marin, shell, algal, settlement, bed |
| Forest Ecology | tree, plot, canopi, height, densiti, seedl, cm, light, stem, gap, diamet, shade, measur, structur, branch |
| Macroecological Data | spatial, distanc, scale, distribut, pattern, movement, cell, locat, cluster, point, aggreg, grid, random, direct, space |
| Monitoring Biodiversity | site, sampl, abund, survey, detect, estim, record, transect, collect, observ, year, count, locat, total, densiti |
| Plant Biology | plant, biomass, leaf, growth, root, leav, product, shoot, nutrient, respons, grow, aboveground, fertil, water, total |
| Macroecology | communiti, divers, rich, variabl, abund, relationship, function, composit, correl, pattern, group, sampl, variat, environment, scale |
| Policy and Conservation | ’s, countri, human, environment, develop, intern, product, year, world, china, scienc, environ, nation, sourc, global |
| Trait Evolution | trait, select, fit, variat, evolut, phenotyp, environ, adapt, evolutionari, environment, plastic, correl, respons, evolv, genet |
| Remote Sensing | urban, map, cover, land, road, imag, develop, veget, class, region, remot, land_cover, type, human, satellit |
| Migration (Birds) | bird, breed, territori, migrat, popul, year, pair, migratori, winter, avian, season, migrant, habitat, owl, forag |
| Phenology | temperatur, season, year, period, winter, summer, annual, spring, month, day, condit, variat, variabl, rainfal, date |
| Herbivory and Plant Pests | plant, herbivor, insect, damag, herbivori, parasitoid, aphid, beetl, host, feed, resist, spider, defens, arthropod, interact |
| Insect Ecology | coloni, ant, larva, larval, bat, butterfli, fli, worker, forag, develop, insect, emerg, flight, queen, wing |
| Assessing and Managing Extinction Risk | conserv, protect, reserv, biodivers, protect_area, habitat, threaten, extinct, plan, threat, assess, list, popul, manag, region |
| Soil Science | soil, litter, microbi, decomposit, organ, miner, sampl, rate, fungi, fungal, bacteri, root, activ, content, communiti |
| Evolutionary Stable Strategies | cost, valu, scenario, strategi, model, optim, effici, requir, energi, benefit, decis, resourc, assum, reduc, alloc |
| Marine Ecology | sea, marin, coastal, ice, ocean, turtl, coast, bay, seal, water, island, beach, gulf, atlant, shark |
| Conservation and Society | manag, conserv, local, polici, develop, resourc, social, communiti, govern, project, plan, environment, peopl, research, particip |
| Themes in Ecology | ecolog, fig, ecol, scienc, swedish, tabl, non, signific, long, sci, academi, field, biol, first, term |
| Plantations | forest, stand, pine, tree, wood, harvest, oak, spruce, boreal, year, manag, age, log, birch, type |

**S4**: Determining the field–specific bias of Topics by the difference of their median log10(probability) of occurrence in ecology (ec) papers and conservation biology (cb) papers.

| topic | | median log10($p$) ec | | median log10($p$) cb | | difference | |
| --- | --- | --- | --- | --- | --- | --- | --- |
| **ecology–biased** | |  | |  | |  | |
| Trait Evolution | | -2.23 | | -4.89 | | 2.67 | |
| Statistical Inference | | -1.67 | | -4.13 | | 2.41 | |
| Community Ecology | | -2.60 | | -4.81 | | 2.21 | |
| Experimental Ecology | | -2.58 | | -4.65 | | 2.07 | |
| Population Ecology | | -3.07 | | -4.56 | | 1.48 | |
| Macroecology | | -3.08 | | -4.52 | | 1.45 | |
| Trophic Interactions | | -4.33 | | -4.88 | | 0.48 | |
| Macroecological Data | | -4.15 | | -4.46 | | 0.31 | |
| Macroevolution | | -4.68 | | -4.87 | | 0.20 | |
| Metapopulation Ecology | | -4.62 | | -4.82 | | 0.20 | |
|  | |  | |  | |  | |

| **conservation biology–biased** | |  | |  | |  | |
| --- | --- | --- | --- | --- | --- | --- | --- |
| National Parks | | -1.41 | | -4.96 | | -3.55 | |
| Conservation and Society | | -5.03 | | -1.92 | | -3.11 | |
| Policy and Conservation | | -4.99 | | -2.28 | | -2.71 | |
| Land-Use Change | | -5.00 | | -3.28 | | -1.72 | |
| Human Impacts On Tropical Forests | | -4.92 | | -3.97 | | -0.93 | |
| Physical Properties of Rivers | | -4.90 | | -3.99 | | -0.90 | |
| Behavioural Ecology | | -4.74 | | -4.02 | | -0.73 | |
| Remote Sensing | | -4.95 | | -4.25 | | -0.71 | |
| Grassland Ecology | | -4.78 | | -4.16 | | -0.62 | |
| Evolutionary Stable Strategies | | -4.79 | | -4.25 | | -0.53 | |

**S5**: Determining the field–specific bias of Topics by relative risk (RR) of occurrence in discretized Topics. Count ec is the number of times a given ecology–biased Topic appears in papers belonging to either field; count cb the number of times a given conservation biology–biased Topic does. RR is calculated as the probability that a Topic is found in ecology relative to being found conservation biology

| topic | | count ec | | count cb | | RR | |
| --- | --- | --- | --- | --- | --- | --- | --- |
| **ecology –biased** | |  | |  | |  | |
| Trait Evolution | | 3933 | | 281 | | 1.08 | |
| Community Ecology | | 2972 | | 303 | | 0.95 | |
| Macroevolution | | 3168 | | 571 | | 0.68 | |
| Trophic Interactions | | 2909 | | 649 | | 0.63 | |
| Epidemiology | | 1292 | | 348 | | 0.60 | |
| Sexual Selection | | 1971 | | 461 | | 0.60 | |
| Herbivory and Plant Pests | | 1812 | | 420 | | 0.56 | |
| Plant Biology | | 2259 | | 617 | | 0.54 | |
| Metapopulation Ecology | | 3224 | | 943 | | 0.50 | |
| Experimental Ecology | | 4636 | | 1384 | | 0.49 | |
|  | |  | |  | |  | |

| **conservation biology –biased** | |  | |  | |  | |
| --- | --- | --- | --- | --- | --- | --- | --- |
| Conservation and Society | | 742 | | 5493 | | -0.95 | |
| National Parks | | 839 | | 6846 | | -0.94 | |
| Policy and Conservation | | 632 | | 4168 | | -0.83 | |
| Land-Use Change | | 273 | | 2075 | | -0.81 | |
| Hunting and Human-Wildlife Conflict | | 576 | | 2427 | | -0.73 | |
| Human Impacts On Tropical Forests | | 715 | | 2301 | | -0.57 | |
| Remote Sensing | | 462 | | 1630 | | -0.56 | |
| Physical Properties of Rivers | | 546 | | 1432 | | -0.48 | |
| Marine Ecology | | 493 | | 1420 | | -0.48 | |
| Plantations | | 909 | | 1491 | | -0.30 | |

**S6**: This heatmap shows the difference between the independently calculated kernel density estimation in t–SNE space for conservation biology and ecology. Negative values imply an area of greater interest to conservation biology, positive values imply an area of greater interest to ecology.

**S7**: In order to model how biased topics — those favoured by ecology or conservation biology — evolved in both fields, we extracted the Topic probabilities of the ten most conservation biology– and ten most ecology–biased Topics for all papers (Table 3), giving us 20 probabilities per paper or 642,080 probabilities in all. Each probability was identified by: (i) the year in which the paper was published (“year”); (ii) the field in which the paper originated (“field”); (ii) whether it belonged to an ecology– or conservation biology–biased Topic (“bias”). We then modeled the effect of these factors on the median log10(probability) of the combined Topic distribution using quintile regression, and found that all interaction terms were highly significant.

| coefficient | value | std. error | t-value | $P$ |
| --- | --- | --- | --- | --- |
| intercept | -66.20581 | 10.47059 | -6.32302 | 0.00000 |
| field(ecology) | 88.28454 | 10.48612 | 8.41918 | 0.00000 |
| bias(ecology) | 73.65200 | 10.61373 | 6.93931 | 0.00000 |
| year | 0.03159 | 0.00521 | 6.06494 | 0.00000 |
| field(ecology):  bias(ecology) | -257.68780 | 23.33802 | -11.04155 | 0.00000 |
| field(ecology):  year | -0.04505 | 0.00522 | -8.63506 | 0.00000 |
| bias(ecology):  year | -0.03765 | 0.00528 | -7.12961 | 0.00000 |
| field(ecology):  bias(ecology):  year | 0.13027 | 0.01161 | 11.21630 | 0.00000 |

**S8**: Counts of papers in seven ecology journals and nine conservation biology journals, 2000-2015. **A.** “cb" represents conservation biology; “ec” ecology. Cumulative counts. Lines are 2nd order polynomial fits by least-squares regression. **B.** Annual counts. Lines are linear fits by least squares regression. ecology: slope $=21.34\pm2.97$ SE, $r^{2}=0.8$; conservation biology: slope $=32.95\pm5.33$ SE, $r^{2}=0.75$.

**S9**: Counts of conservation biology papers containing a given ecology-biased Topic with a probability $\geq0.05$. Of the ten Topics, eight show little change over time or a decline after 2005. Only “Community Ecology” and “Trophic Interactions” increase, keeping roughly pace with the increased rate of publication in the field as a whole. Trends are illustrated with Loess smoothers.


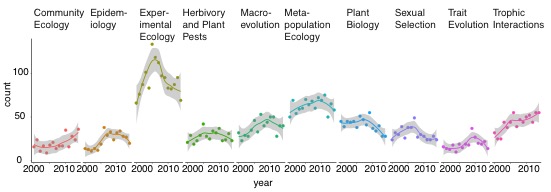


**S10:** a replotting of figure 6 panels F-I from the main text with unified scale on the y-axis across all panels.
